# Direct to Public Peer Support and e-Therapy Program Versus Information to Aid Self-Management of Depression and Anxiety: Protocol for a Randomized Controlled Trial

**DOI:** 10.2196/resprot.8061

**Published:** 2017-12-18

**Authors:** Catherine J Kaylor-Hughes, Mat Rawsthorne, Neil S Coulson, Sandra Simpson, Lucy Simons, Boliang Guo, Marilyn James, Paul Moran, Jayne Simpson, Chris Hollis, Anthony J Avery, Laila J Tata, Laura Williams, REBOOT Notts Lived Experience Advisory Panel, Richard K Morriss

**Affiliations:** ^1^ National Institute for Health Research: Collaboration for Leadership in Applied Health Research and Care East Midlands University of Nottingham Nottingham United Kingdom; ^2^ Division of Rehabilitation and Ageing School of Medicine University of Nottingham Nottingham United Kingdom; ^3^ Nottinghamshire Healthcare NHS Foundation Trust Institute of Mental Health Nottingham United Kingdom; ^4^ National Institute for Health Research: MindTech Healthcare Technology Co-operative Institute of Mental Health Triumph Road Nottingham United Kingdom; ^5^ Centre for Academic Mental Health School of Social & Community Medicine University of Bristol Bristol United Kingdom; ^6^ Division of Primary Care School of Medicine University of Nottingham Nottingham United Kingdom; ^7^ Division of Epidemiology & Public Health School of Medicine University of Nottingham Nottingham United Kingdom

**Keywords:** depression, anxiety, peer support, online, self-management

## Abstract

**Background:**

Regardless of geography or income, effective help for depression and anxiety only reaches a small proportion of those who might benefit from it. The scale of the problem suggests a role for effective, safe, anonymized public health–driven Web-based services such as Big White Wall (BWW), which offer immediate peer support at low cost.

**Objective:**

Using Reach, Effectiveness, Adoption, Implementation and Maintenance (RE-AIM) methodology, the aim of this study was to determine the population reach, effectiveness, cost-effectiveness, and barriers and drivers to implementation of BWW compared with Web-based information compiled by UK’s National Health Service (NHS, NHS Choices Moodzone) in people with probable mild to moderate depression and anxiety disorder.

**Methods:**

A pragmatic, parallel-group, single-blind randomized controlled trial (RCT) is being conducted using a fully automated trial website in which eligible participants are randomized to receive either 6 months access to BWW or signposted to the NHS Moodzone site. The recruitment of 2200 people to the study will be facilitated by a public health engagement campaign involving general marketing and social media, primary care clinical champions, health care staff, large employers, and third sector groups. People will refer themselves to the study and will be eligible if they are older than 16 years, have probable mild to moderate depression or anxiety disorders, and have access to the Internet.

**Results:**

The primary outcome will be the Warwick-Edinburgh Mental Well-Being Scale at 6 weeks. We will also explore the reach, maintenance, cost-effectiveness, and barriers and drivers to implementation and possible mechanisms of actions using a range of qualitative and quantitative methods.

**Conclusions:**

This will be the first fully digital trial of a direct to public online peer support program for common mental disorders. The potential advantages of adding this to current NHS mental health services and the challenges of designing a public health campaign and RCT of two digital interventions using a fully automated digital enrollment and data collection process are considered for people with depression and anxiety.

**Trial Registration:**

International Standard Randomized Controlled Trial Number (ISRCTN): 12673428; http://www.controlled-trials.com/ISRCTN12673428/12673428 (Archived by WebCite at http://www.webcitation.org/6uw6ZJk5a)

## Introduction

### Background

Unipolar depression and anxiety are the second and seventh leading causes of years lived with disability in the world among all health problems, respectively, according to the World Health Organization [1]. The 12-month point prevalence of unipolar depression, anxiety disorder, and mixed anxiety and depression is 15% in the United Kingdom [2] and ranges from 4% to 20% across 14 different developed and nondeveloped countries in the world [3]. There are a number of reasons why a population approach [4] to the management of depression and anxiety employing supported self-management is required: (1) the scale of the problem is too great both in terms of prevalence, recurrence rates, and access to be met through primary care and secondary care services alone, with only 33% of people receiving any treatment in the United Kingdom with broadly similar rates in developed countries in Europe and North America and half these rates in less developed countries [3,5]; (2) people may choose not to seek professional help for a number of reasons, including a preference for self-management, fear of stigma, fear or lack of motivation resulting from anxiety and depression, or as a result of having a previous bad experience of mental health care [6,7]; (3) people may be unsure where the boundary lies between their experience of stress on the one hand or clinical anxiety and depression on the other [6], sometimes preferring guidance before, or instead of, seeking medical care; (4) people may prefer to manage their problems themselves (personal empowerment) and at their own pace but seek guidance and support when they choose [8,9]; (5) to combat social isolation, people with depression and anxiety may sometimes prefer to develop a social network of people who are sympathetic to and understand what it is like to have depression, anxiety, or related mental health problems [10,11]; and (6) primary and secondary care services largely manage acute symptoms of anxiety and depression, focusing particularly on depression as a common cause of suicidal behavior and may not provide sufficient information and support for self-help to prevent recurrence [12]. Public health interventions to manage very common problems such as cigarette smoking, weight loss, and diabetes care have increasingly utilized the Internet to reach the public [13]. Provided safeguards are put in place to identify and transfer care to primary and secondary care services when appropriate (eg, high suicide risk), Internet programs offering a range of tailored information and support might address all or some of the six scenarios outlined above, among others.

Big White Wall (BWW) is a well-established digital service (website and apps) being accessed by approximately 13,000 people in the United Kingdom in the past year. Currently, BWW has been purchased by the armed forces, some universities, and 25% of clinical commissioning groups throughout England that provide free access to 98% of the users. Only 2% of the users are individuals who pay for the service through a £25 monthly subscription. It is also available or being piloted in the United States, Canada, Australia, and New Zealand.

BWW offers the following to people over the age of 16 years [14]: (1) Web-based assessment to assess common mental health problems and comorbid physical conditions; (2) moderated online peer support network: a community of peers, professionally staffed at all times, enabling safe, anonymous support through talking therapies and creative self-expression; (3) guided support: a range of self-managed and facilitated programs for individuals and groups for depression and anxiety and related issues such as sleep, smoking, and alcohol problems based on cognitive behavioral therapy (CBT) and social support principles; and (4) live therapy: a range of real-time therapies by instant text, audio, or video from a panel of approved BWW therapists offering CBT, interpersonal therapy, person-centered counseling, or integrative counseling.

On the basis of public health principles, BWW emphasizes a recovery model to improve well-being and is theoretically based on a social model of depression emphasizing autonomy, hopefulness, and support [14] (see [15] and [16] for reviews of the social model of depression). There are no waiting lists, eligibility criteria, or restricted opening hours (available 24/7). Specially trained counselors employed by BWW as *wall guides* facilitate interactions, ensuring that a culture of respect toward others, tolerance, mutual learning, and safety are maintained at all times. Mental well-being is seen as a systemic interplay of factors that contribute to an individual’s engagement with themselves, their social networks and communities, and the society in which they live. Mental well-being is also seen as being intimately connected with physical health and general well-being.

BWW offers guided self-help based on CBT principles, with additional peer support—interventions that are recommended as face-to-face interventions for mild to moderate depression by the National Institute for Health and Care Excellence (NICE) in England and Wales [12]. However, the randomized controlled trial (RCT) evidence base for such interventions using a peer social support model requires more extensive testing. Currently, there is no RCT evidence that BWW is effective and only one large previous RCT of Internet peer support in 311 people with primary depression and anxiety [17]. This showed that both Internet peer support and Internet peer support plus guided self-treatment of depression (both of which BWW offers) versus information about depression and anxiety alone, improved depression and empowerment with additional benefits on quality of life (QOL) and self-esteem in the combined intervention [17,18]. The effects on empowerment were immediate, although improvement in QOL was only apparent at 6 months. Peer support was utilized more extensively than a formal course of CBT. Furthermore, the study found that weekly contact with the site was associated with greater improvements on ratings of social support and loneliness. However, it is not a test of the reach or effectiveness using the Reach, Effectiveness, Adoption, Implementation (including economics) and Maintenance (RE-AIM) methodology of a peer support program backed by a public health campaign, unlike this study.

Moreover, BWW is a complex intervention that operates differently to a psychological intervention in that people choose exactly when and how they utilize it rather than commit themselves to a course of treatment of a defined duration and frequency, which may be restricted by external issues such as clinician availability.

Peer support Internet interventions take less effort for service users than Internet-guided CBT [18]. The involvement is usually ongoing and unrelated to psychiatric crisis, for example, suicide or self-harm, unlike crisis lines such as Samaritans. In principle, relief from depression or anxiety can be achieved through BWW by improving the quality and consistency of support [19]. The effectiveness of BWW may be in keeping with the social model of depression and anxiety on which it was conceived. The onset of depression and anxiety is precipitated by the self-perception of a potential or actual lasting and severe threat to the person's well-being from a life event or a life difficulty in the absence of social support (isolation) or insufficient social support [20]. Relief from depression or anxiety is achieved by improved quality and consistency of support or life events that offer fresh starts or increased security. People with personality dysfunction may obtain less relief from depression and anxiety but still benefit [21].

Thus, a person with depression and anxiety has access through BWW to appropriate psychosocial support when they require it. They are given guidance and are enabled to choose to intervene in their own mental well-being (empowerment). This early intervention approach may preempt the need for later intervention if the person becomes worse. Although guided, people are encouraged to make their own decisions on how to use BWW so they retain their autonomy because perceived control is thought to improve outcome in depression and anxiety [15,16].

Alternatively, the National Health Service (NHS) has developed a free website providing information on mental health conditions and locally available resources called NHS Moodzone. It does not provide anonymized moderated peer support.

The RE-AIM (model [22]), which is designed to enhance the quality, speed, and public health impact of translational research in a defined population, will be used as a framework for the study. Here it will be used to compare free access to BWW versus free access to information about depression and anxiety from the NHS Choices Moodzone website for people who score above depression or anxiety caseness in one area of England serving inner city, urban and rural areas, and where there is no current institutional or commissioned access to BWW.

The overall aim was to utilize a strategy that is broadly similar to how BWW is usually implemented in a geographical area, with clinical champions from primary care and service users alongside publicity through local public health campaigns and commissioners.

### Objectives

The objectives for this study (known as “the REBOOT study”) are bound within the RE-AIM framework:

Reach: To determine the number and representativeness of participants invited and eligible to receive BWW or the NHS Choices Moodzone website compared with the expected number of participants (based on estimates from census data in the study area).

Effectiveness: To determine the short-term clinical effectiveness of randomization to BWW versus the Moodzone website on well-being (primary outcome), depression symptoms, anxiety symptoms, social function, and QOL in one locality over 3 months.

Adoption (by services): To determine the number, percent, and representativeness of NHS primary care practices and organizations, secondary care mental health, community and acute trust, third sector, and social care organizations that referred people to either BWW or the Moodzone website.

Implementation: To explore the implementation of the BWW program, including barriers and drivers to reach, effectiveness and adoption, and an economic evaluation of its costs and cost-effectiveness from personal, social, and health care perspectives.

Maintenance: To determine (1) The maintenance of treatment effects on well-being, depressive symptoms, anxiety symptoms, QOL, and social function over 6 months and (2) The take-up by organizations and implementation (number, percentage of BWW across the East Midlands) after the trial has been completed.

We will also explore how the interventions may work by quantitatively examining moderators and mediators of outcome and conducting a qualitative analysis, and in the case of the BWW intervention, discourse analysis.

The discourse analysis aims to answer the following research questions:

- How social support is provided within peer support exchanges within BWW?
- What topics are discussed by trial participants when using BWW?

## Methods and Analysis

### Trial Design

A single-blind, pragmatic RCT will be conducted in the county of Nottinghamshire, United Kingdom using a fully automated bespoke study website. Eligible participants, recruited through self-referral methods such as social media, general practitioner (GP) advertisements, and general marketing will be allocated at random and without stratification to receive either 6 months free access to BWW or be signposted to the NHS Moodzone website. The primary outcome will be clinical effectiveness (mental well-being) of BWW versus Moodzone as measured by the 14-item Warwick-Edinburgh Well-being Scale [23,24] from baseline, 3 to 6 weeks. Maintenance of effect at 12 and 26 weeks will be secondary outcome measures.

This RCT forms part of the East Midlands Collaboration for Leadership in Applied Health Research and Care (CLAHRC-EM), an applied health care research center funded by the National Institute for Health Research (NIHR), the Universities of Nottingham and Leicester, and over 50 partners from health care, social care, and industry across the East Midlands. Funded across the United Kingdom, there are 13 CLAHRCs that focus on the clinical and cost-effectiveness of translational research that has been identified as priority areas by local services. Service delivery studies of this kind, therefore, require more than just the clinical outcomes that traditional RCTs might produce and present the need to explore cost-effectiveness, risk, adoption by services, reach, and the barriers and drivers to implementation.

Ethical approval has been granted by the Local Research Ethics Committee (REC 16/EM/0204), and final approval was received from the Health Research Authority.

### Recruitment

To optimize recruitment to the trial as well as the regional uptake of BWW, a public health engagement campaign will run alongside participant recruitment via local services to maximize the opportunities for people without, as well as with current access to mental health services, to take part in the trial. Potential participants will all self-refer to the study website after accessing information about the study via two main routes: (1) general media and digital social media advertising and (2) recommendation from health care professionals and other support workers in NHS primary care, NHS secondary care, social care, and third sector and community services. Recruitment and adoption throughout the study will be closely monitored and recorded in order that networks and reach can be determined and analyzed.

### Engagement Strategy

The public engagement strategy and recruitment to the study will run in parallel with each other. The engagement strategy will, for 1 month, publicize the study to NHS, local authority, and third sector organizations in Nottinghamshire a month before recruitment to the trial begins. The study will be integrated into the early intervention stream of the Clinical Commissioning Groups and Health and Well-being Board Mental Health Strategy for Nottinghamshire. A range of methods will be used to engage with potential study participants, including:

- Identification of primary care practices and other groups of health professionals, for example, health visitors and community pharmacists that are known to have an interest in mental health and a willingness to adopt new interventions early after their introduction in one part of the county before moving to other parts
- Introducing a local general media advertising campaign (eg, bus and tram adverts, newspapers, and radio), followed by a Facebook and social media campaign
- Presenting the study to interested community groups, targeting both the young (eg, further education colleges and universities) and old (eg, Age UK), vulnerable groups (eg, new parents living in disadvantaged areas, through Surestart), third sector mental health groups (eg, Mind branches in Nottinghamshire), and local organizations promoting self-help (eg, Self-Help Nottingham)
- Producing online and offline presentations and written materials
- Other novel approaches as they might arise in public health and primary care, for example, public health message with receipts in high footfall supermarkets or shopping centers

Patient and public involvement is central to the design and delivery of this study. A user consultant has been appointed to develop novel approaches and monitor, iterate, and evaluate the effectiveness of the different engagement methods, with an action-research type approach. The user consultant will develop a lived experience advisory panel to help shape the study process throughout. They will work with at least one GP knowledge broker (who will act as clinical champions for the study as a whole) in other areas where BWW has been implemented successfully; this role has been important linking with GP and other community health professionals and the early intervention stream. Regular checks will show which methods are best for broadening the reach of the study and BWW to a wider range of people and groups and especially those isolated.

On the basis of 6- to 12-month engagement strategies using a part-time engagement officer carried out by BWW in the West Midlands (Dudley, Wolverhampton, and Walsall; in total a similar size population to that of Nottinghamshire), 1950 participants (1.6% of the population) were recruited. We anticipate a similar rate of recruitment in Nottinghamshire, with approximately 2200 participants recruited to our study website over a 12-month recruitment period, and we will have the additional help of the user consultant and the public health campaign publicizing the study.

The learning achieved from the recruitment period will inform the best approaches to engage with the public in later stages of the study. A feedback conference will engage with study participants and other stakeholders groups (commissioners, Improving Access to Psychotherapy services, public health, etc) to enable understanding of the wider validity and relevance of emerging findings. An effective public dissemination strategy, informed by our earlier learning, will communicate the findings of the research to a wide audience to help mobilize the new knowledge as part of the implementation strategy (see later section).

### Sample and Eligibility

Potential participants from the county of Nottinghamshire will be able to self-refer, and their eligibility will be assessed by an automated digital program on the study website. The study website requests GP practice contact details in case the participant is ineligible for the study.

The inclusion criteria are as follows:

- Aged 16 years or over
- Resident in the County of Nottinghamshire
- Scores between 10 and 20 on the 9-item Personal Health Questionnaire (PHQ-9) [25] and/or 10 or more on the 7-item Generalized Anxiety Disorder (GAD-7) questionnaire [26], indicating probable caseness for depression and anxiety, respectively, but not a definite diagnosis of depression or anxiety disorder
- Access to the Internet through a computer, tablet, or smartphone (Windows, iPhone operating system [iOS, Apple Inc], and Android)
- Able and willing to give informed consent (through electronic consent)

The exclusion criteria are as follows:

- Scores 21 or more on the 9-item Personal Health Questionnaire (PHQ-9, severe depression)
- Scores 2 or 3 on PHQ-9 item “thoughts that you would be better off dead or of hurting yourself in some way.”
- Scores below 10 on PHQ-9 and 7-item Generalized Anxiety Disorder (GAD-7) questionnaire
- BWW and Moodzone are only available in English. Therefore, the website will recommend to participants that if they do not feel that they are sufficiently proficient in the use of the English language, they need not take part. There will be no test of proficiency.

Participants who are ineligible for the trial because they score in the severe range on the PHQ-9 or signs of suicidality will be provided with an opportunity to request that the study team inform their GP, mental health care team, or carer of their current mood state. If they choose not to take this offer, the study team will follow this up on one occasion with an email to ask them again if they would like the team to inform their GP or care team. If they do not reply, the team will consider this confirmation that they do not wish us to act on their behalf.

Participants that are under age will be given details of local children’s mental health services. They will be informed that they may return to the study website if they turn 16 years within the recruitment period, should they still wish to participate.

Information for participants and the associated consent forms are provided electronically within the study website. Participants who wished to discuss the study could email and telephone the study team if they had any further questions before consenting to the study. An email confirming consent is sent to a participant once they have fully enrolled in the study.

### Expected Duration of Participant Participation

**Figure 1:**
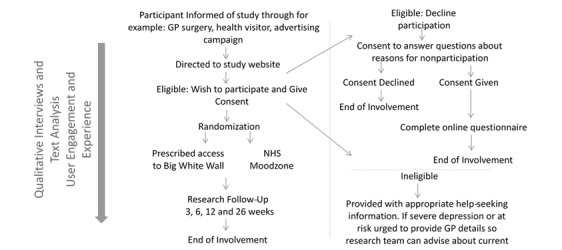
Participants' journey through the study.

Participation in the study will be for 6 months (see Figure 1 for participant journey through the study). Participants will receive electronic follow-up invitations at 3, 6, 12, and 26 weeks after randomization to be completed on the website. Participants may also be asked to take part in a short interview by phone or face-to-face to talk about their experiences of services and/or the study no later than 3 months from the ending of their participation.

### Interventions

#### Randomization: Arm 1—BWW

Participants allocated to receive 6 months free access to the BWW website will create a user profile using a pseudonym that will be linked to the trial identification that they are assigned within the study website. Participants will be able to access any part of the BWW site (apart from the option of personalized therapy or counseling sessions that have to be prescribed by a clinician [ie, is not offered direct to the general public], and for this reason it will not be accessible for participants in this study) and interact with other users within the boundaries of the site’s house rules. [14]. Anonymized records of log-ins, time on site, interactions, and page categories will be recorded by BWW on behalf of the study team.

#### Randomization: Arm 2—Participants Allocated to Moodzone

Participants will be directed to the Moodzone area of the NHS Choices website, a free to access website providing information on mental health conditions and locally available resources. Participants will be able to access all of the available material on mental health, including depression and anxiety. The site contains reading material and suggestions about maintaining mental health and provides measures of depression and anxiety for visitors to use.

All participants will be asked about whether they have received or used any additional mental health resources during their time in the study. NHS Moodzone access is used as the control digital resource so that all the participants are offered some help for their problems with depression or anxiety, but the control group do not access moderated, anonymized peer social support.

### Outcome Measures

Once consented, participants will be asked to complete self-rated questionnaires to measure well-being, depression, anxiety, work and social adjustment, QOL (for economic analysis), receipt of services (for economic analysis), social support, and personality dysfunction (see Table 1). These will be completed online (through the study website) in approximately 20 to 30 min, with the primary outcome data collected first. All data will be stored on the website and downloaded and anonymized by the clinical trials manager.

#### Primary Outcome Measure

- Change in self-rated well-being at from baseline to 3 and 6 weeks after baseline using the 14-item Warwick-Edinburgh Mental Well-Being Scale (WEMWBS)

#### Secondary Outcomes Measures

- Maintenance of well-being 12 and 26 weeks using the WEMWBS
- GAD-7 scale—completed as part of eligibility at baseline and then at 3, 6, 12, and 26 weeks as a brief measure of the severity of 7 symptoms of anxiety
- PHQ-9—completed as part of eligibility at baseline and then at 3, 6, 12, and 26 weeks as a measure of depression severity according to the *Diagnostic Statistical Manual, 4th edition* criteria [27]
- 12-item Medical Outcomes Study Short Form health survey version 2.0 (SF-12v2) [28]—a short and practical measure of health-related QOL derived from the longer SF36 that is obtained during interview
- Social support is measured using the 8-item Medical Outcomes Study Social Support Survey (MOS-SS) [29], and it is completed at baseline
- Social function on the 8-item Work and Social Adjustment Scale (WSAS) [30]—a simple measure of impact on function, which is attributable to a particular cause such as depression or anxiety. It is completed at baseline and then at 3, 6, 12, and 26 weeks

Mediators of the effectiveness of BWW compared with Moodzone on mental well-being might be the emotional and informational support subscales of the MOS-SS [29]. Data collected by BWW suggest that perceived social support might be an important mediator of outcome, particularly in people experiencing life events and loneliness.

Moderators of outcome at baseline might be the presence of life events in the previous 6 months, measured by the 12-item Brugha Inventory of Life Events [31], and the presence of anxiety alone at baseline. Personality dysfunction will be measured at baseline using the 8-item Standardised Assessment of Personality-Abbreviated Scale (SAPAS) [32]. There may be an interaction between the presence of life events at baseline and greater perceived emotional support from BWW at 3 months and on mental well-being at 6 months, whereas higher scores on the SAPAS may predict dropout and little difference between the treatment arms on mental well-being at 6 weeks.

**Table 1:** Timing and delivery of outcome measures.

Measure	Baseline	3 weeks	6 weeks	12 weeks	26 weeks
WEMWBS^a^	Website	Website	Website	Website	Website
PHQ-9^b^	Website	Website	Website	Website	Website
GAD-7^c^	Website	Website	Website	Website	Website
WSAS^d^	Website	Website	Website	Website	Website
MOS-SS^e^	Website	X^f^	X	X	X
SAPAS^g^	Website	X	X	X	X
Brugha Life^h^	Website	X	X	X	X
SF12-v2^i^	Website	X	X	X	Interview
CSRI^j^	Website	X	X	X	Interview

^a^WEMWBS: Warwick-Edinburgh Mental Well-Being Scale.

^b^PHQ-9: 9-item Personal Health Questionnaire.

^c^GAD-7: 7-item Generalized Anxiety Disorder Scale.

^d^WSAS: Work and Social Adjustment Scale.

^e^MOS-SS: 8-item Medical Outcomes Study Social Support Survey.

^f^X: Measures are not collected at this time point.

^g^SAPAS: Standardised Assessment of Personality-Abbreviated Scale.

^h^Brugha Life: 12-item Brugha Inventory of Life Events.

^i^SF-12v2: Medical Outcomes Study Short Form health survey version 2.0.

^j^CSRI: Client Service Receipt Inventory.

To explore the reach, adoption, and implementation arms of the RE-AIM framework, a number of process measures will be recorded as the study progresses:

- Live log of services, individuals, groups, and charities that are approached to engage with the study. The networks that surround these and how this may spread will be recorded and used to determine reach and adoption. A network of practice comprising all health care stakeholders will be established to explore implementation pathways.
- Reach will be further explored using Internet analytic software or to derive anonymous data around hit and bounce rates, pages visited, length of visit, type of geography, and simple demographics to the study website can be recorded, downloaded, and analyzed
- Implementation will be explored when highlighting the barriers and drivers to reach, effectiveness, and adoption assessed through qualitative work with the services, groups, and individuals engaged through recruitment, as well as an economic evaluation of its costs and cost-effectiveness from personal, social, and health care perspectives. In particular, we will explore how participants utilize BWW in relation to the literature on similar websites offering peer support and information for physical illness and literature on social and organizational aspects of depression and anxiety care.
- Working with the local Academic Health Science Network to record the take-up by organizations and implementation (number, percentage, and representativeness in East Midlands) of BWW across the East Midlands after the trial has been completed should it prove to be clinically and cost effective.

In terms of socioeconomic inequalities, we will record the following provided such detailed recording of information does not deter participation: postcode, age, gender, ethnicity marital status, highest level of education, and employment.

### Sample Size and Justification

Given that the focus of BWW is on improving mental well-being rather than specifically depression or anxiety, a clinically important difference on the WEWBS was selected as the primary outcome. Data from BWW online support groups show clinically important differences in depression by 6 weeks, so the primary outcome will be a change in the WEWBS from baseline to 3 and 6 weeks. The minimal clinically important difference (MCID) for adults on the 14-item WEWBS with mild to moderate depression and anxiety is 3 points and WEWBS scores are normally distributed [24]. On the basis of data from a Web-based CBT intervention versus information from an RCT in a similar participant group with a similar design [33], and inflating the variance (to allow for contamination) at 6 weeks by almost 50%, we estimate the sample size required to detect a 3-point MCID between the BWW and Moodzone groups at 6 weeks to be 676 in total or 338 per treatment group using the analysis of covariance method with 90% power to show a difference at two-sided 5% significance level assuming zero correlation between pre- and posttreatment outcomes. In the previous study, the intervention and control groups were 42.20 (standard deviation [SD] 9.83) and 42.32 (SD 9.64), respectively, and by 6 weeks they were 44.46 (SD 8.1) and 41.92 (SD 9.18). In the power calculation, we have assumed that at baseline, each intervention group will have a mean baseline score of 42.20 (SD 9.83) and that this will increase to 44.46 (SD 12.0) in BWW and fall slightly to 41.46 (SD 12.0) in the Moodzone control group at 6 weeks. There are no data currently available using the WEWBS with BWW, but BWW online support groups show a drop in PHQ-9 score from 13.9 (SD 7.1) to 8.6 (SD 6.5) at 6 weeks—a change that is equal to an MCID of 5.0 points suggesting that BWW can produce clinically important differences in outcome.

The analysis will use multilevel modeling because of repeated measures in the same individuals. People directly participate in the study so there is no other form of clustering operating in this study. Our power calculation performed has already taken into consideration clustering because of repeated measures. Assuming a 50% loss to follow-up, a total of 1352 participants are required for the RCT [33,34] but typically BWW would be expected to enroll 2200 people in a county the size of Nottinghamshire if the uptake of BWW was consistent with the general pattern seen in similar parts of the country. Furthermore, in a study with a similar design [33], 3070 (63.52%, 3070/4833) participants who were enrolled into the study completed eligibility criteria on a website and were invited to take part. Taking a conservative approach, we would recruit approximately 1400 participants out of 2200 people meeting eligibility criteria and enrolling onto the study site. This would be sufficient to recruit an adequate sample size. Stata 14 (StataCorp LLC, USA) was used to perform power analysis [35].

### Randomization and Monitoring

The treatment to which a participant is assigned will be determined by a computer-generated pseudorandom code using random permuted blocks of varying size by a randomization system embedded within the website. No stratification or minimization is required. The outcome will be single-blind with the clinical trials manager and research associates responsible for the collection, cleaning, and analysis of the data remaining blind to arm allocation until data collection has been completed. Cases of unblinding will be recorded electronically but will not be excluded. Unblinding will only be necessary upon completion of data collection.

Overall, trial monitoring and oversight will be carried out by the CLAHRC-EM Scientific Committee who will act as the trial steering committee and data monitoring committee. The CLAHRC-EM scientific committee is composed of independent experts in statistics, epidemiology, medicine, and patient and public involvement. They will be sent quarterly reports on the status of the study and have the power to recommend or implement changes to the protocol if necessary. They are also able to stop the study if it is deemed unsafe or is failing to recruit after all avenues to recruitment have been exhausted.

There is no planned assessment of safety within the study design, and unless participants report any intercurrent illness or adverse events directly to the study team, it is unlikely that these can be recorded systematically.

### Statistical Analysis

Analysis of the primary and secondary outcome measure data will be carried out by the trial statistician, who will remain blind to arm allocation, using STATA 14.

In a RE-AIM study, all outcomes are considered to address important facets of a public health intervention. Therefore, we will examine reach (the percentage and representativeness) of participants entering the trial. Exploratory analysis will summarize outcome variables and participant background variables by treatment arms across follow-up time with mean (SD) for normally distributed data, median (interquartile range) for skewed variables, and frequency (percentage) for observed level of categorical variables. The analysis to examine the treatment effect will be performed on an intention-to-treat basis. As the primary outcome WEWBS score will be repeatedly measured at baseline, 3 weeks, and 6 weeks, multilevel modeling will be performed to quantify the treatment effect with participant as a level 2 unit and baseline measurement as a covariate using Stata 14. Missing values will be imputed using a multiple imputation approach under a missing at random assumption. Path analysis will be used to explore changes in perceived emotional social support and empowerment at 3 weeks as potential mediators for treatment effects on mental well-being at 6 weeks, alongside life events, loneliness, having a partner, gender, age, anxiety without depression, and personality dysfunction at baseline as possible moderators for treatment effect. Such information will provide important information to refine both the targeting and content of BWW to optimize its reach and effectiveness.

A detailed statistical analysis plan setting out full details of the proposed analyses will be finalized before the trial database is locked for final analysis. Stata 14 will be used to perform exploratory data analysis and multilevel modeling, Mplus software (Muthen & Muthen, USA) will be used to perform path analysis [36].

### Adoption and Implementation

Adoption will be examined according to the prevalence of uptake and promotion of the study by primary care, secondary care, social care, and third sector organizations. Implementation will be examined in four ways: (1) a quantitative analysis of patterns of acute use of BWW from baseline to 6 weeks according to clinical and sociodemographic factors; (2) an economic evaluation; (3) long-term effects will also be explored using qualitative interviews and patterns of use of data from the websites. An analysis of barriers and drivers of participants, public and patient groups, and health professionals to reach, adoption, effectiveness, and maintenance of BWW using digitally recorded and thematically analyzed individual qualitative interviews; and (4) textual analysis of written comments made by participants and qualitative interviews to explore the process of self-management, peer support, and organization of care on BWW.

### Health Economics

The study will be conducted from an NHS and societal perspective, which will include cost to the individual but will in addition to clinical outcomes measure the participant's own health status using the SF12-v2 during participant interviews [28]. Thus, the study results will be reported in terms of cost-effectiveness and cost-utility. A detailed resource profile will be established for the intervention versus usual care. The resource profile will include capital costs, for example, the technology, licensing agreements, and assumptions around the length of life of the respective Internet technologies and participant costs in each arm (eg, time accessing the Internet). Economic data will be collected using the economic resource proforma (the Client Service Receipt Inventory [37]), which was piloted successfully in a previous study [38]. Economic data around service use will be collected at baseline, and further economic data, including any time lost from work or usual activities, will be collected during interviews with 200 participants at 12 and 26 weeks who consent to be interviewed. Medication costs will be obtained from the British National Formulary, primary care contacts assigned using community and hospital-based costs from NHS reference costs. Information collected from participants will include any time lost from work or usual activities. An incremental cost-effective ratio and cost-effectiveness acceptability curves will be produced for the BWW versus Moodzone sites. This use of this probabilistic analysis is recommended in NICE guidelines and is economically more useful than classical probability estimates of significance.

### Qualitative Analysis

Qualitative interviewing and analysis will be used to determine the barriers and motivations for participant engagement in the study to inform and further develop the study’s engagement strategy. We also intend to explore participants’ experiences of taking part in the study to gain and understand the motivations for use of online peer support. For those in the BWW arm of the study, this will include patterns and levels of engagement (eg, active user vs “lurker”), negative experiences and beliefs about efficacy, and role in personal empowerment. For those in the Moodzone arm, we will interview participants about negative and positive experiences of receiving just information in relation to managing depression and anxiety. Qualitative interviews will be sought with a maximum variance sample based on sociodemographic factors, scores on baseline clinical measures, and whether or not they are using other health services. Participants will be contacted after they have been in the study for 6 weeks. We will also interview those who did not wish to participate or dropped out but indicated they wished to be contacted to leave feedback.

### Participant Messages

All conversation threads over 6 months in which at least one message has been posted by a trial participant will be retrieved by BWW and sent securely and anonymously to the research team. These will include messages posted by nonstudy participants. However, these messages will not be analyzed; rather they are provided to understand the context of the messages posted by the study participants.

The data generated for this phase will be analyzed using content analysis [39]. This approach is frequently used to quantify theoretical concepts and qualitative data categories in the manifest content of large volumes of textual information and has been successfully employed in previous studies [40-42]. We will address the discourse analysis questions as follows:

How social support is provided within peer support exchanges within BWW?

We propose to employ the Social Support Behavior Code [43] as our guiding theoretical framework. This framework has been used extensively in the analysis of online forum communication (eg, [43]) and provides the means to identify and quantify the presence of five key categories of social support: information support, emotional support, network support, esteem support, and tangible assistance. Our analysis will record separately the number of requests for support as well as the provision of support, by category (above), within the dataset.

What topics are discussed by trial participants when using BWW?

We propose to use thematic analysis to address this research question using previous guidelines [44]. This approach allows the systematic analysis of textual data to identify and describe emergent themes based on patterns within the dataset.

## Discussion

Only 33% of people with depression or anxiety receive any help from health services in England [5] and other developed countries in Europe and North America [3]. Public-facing websites, such as BWW, offering support and help may offer effective help delivered at scale to very large populations. Yet, the effectiveness of BWW has yet to be established, and our study will provide the first rigorous test of this.

The design of such a large automated study in one geographical area poses a number of methodological challenges, and our study design has a number of strengths as well as inevitable weaknesses.

### Strengths

The pragmatic design of the trial means that our estimates of effectiveness and cost-effectiveness are likely to be generalizable to other areas of the United Kingdom and other high income populations as this is a direct to the public study that does not rely on health service infrastructure that varies from place to place.

The public health (RE-AIM) approach of the REBOOT study will help to raise awareness of the possibility of digital intervention with a large group of people with depression and anxiety who are not currently engaged with primary care or mental health services. It will also explore whether it provides additional help to those who are already engaged with these services in relation to immediate moderated anonymized support and digital approaches to socialization and recovery that might be more convenient and approachable than comparable face-to-face approaches. It will provide a great deal of information surrounding the reach and adoption of such a resource and the role it plays in their well-being. We will know the clinical, sociodemographic, and health care service use of participants and visitors to the site who complete baseline information but decide not to be randomized as well as those who utilize the interventions, and completers and dropouts from the study. We will also be able to explore through process evaluation moderators and mediators of mental well-being through both treatment arms and in more detail in the BWW treatment arm by detailed quantitative and qualitative analysis of social messaging through BWW. Therefore, there is the opportunity through such process evaluation to improve the reach or effectiveness of digital interventions such as BWW and also to predict more clearly what the impact of BWW might be in an area outside Nottinghamshire if such services were commissioned [45]. The protocol is ambitious, but the feasibility of conducting a fully mobile randomized clinical trial for depression has been demonstrated recently with the randomization of 626 participants [46].

### Limitations

Therefore, we have carefully coproduced the REBOOT study website with e-mental website developers such as M-Habitat [47] and the lived experience of the Lived Experience Advisory Panel of service users, who have also contributed to the public health campaign, to reflect the experience of people of different ages, gender, and sociodemographic background with personal experience of depression and anxiety. By doing so, we hope that the REBOOT public health campaign and study website will engage and connect with people who have depression and anxiety in the community so that they will enter the study and continue in follow-up. We have also considered issues of intrinsic reward (eg, altruism, motivational statements, and feedback on completion) and extrinsic reward (reminders through text, email, and entry of completers into competitions) to encourage completion of data on the website at each time point [48]. Issues around access to the Internet, ownership of devices, and having access and competence with information technology may also be barriers to the use of the interventions that will be explored through qualitative interviews.

A further complication in the digital study is the issue of contamination. People with depression or anxiety might access BWW or Moodzone independently, although in the county of Nottinghamshire the opportunity to enroll in BWW is limited to access through the Armed Forces as new personal subscription has been suspended for the duration of recruitment and follow-up in the REBOOT study. Only one randomization is possible from each web browser within a 30 day period. Participants may be able to deceive the randomization process through access from a different computer or web browser or by clearing the cookie installed to prevent multiple attempts. Internet protocol (IP) address was not used as a marker to prevent multiple randomizations in case several people might be sharing the same computer. There may also be potential leakage with people who live outside Nottinghamshire using work postcodes that are within the county. Furthermore, there are potential problems with contamination through digital users utilizing alternative sources of digital support and information rather than the study treatment allocated to them. For instance people might join Facebook and offer support to each other even though it is not anonymized, and there is no profession moderation or support. If any of these occurred to a significant extent, it may be difficult to show real improvements in clinical and cost-effectiveness. To understand these potential sources of contamination, we are asking all participants about use of other sites.

The design of studies to evaluate the reach, adoption, clinical-effectiveness, and cost-effectiveness of direct to the public digital health interventions for mental health problems are important, given the reach, popularity, and low cost of such approaches in a world that is increasingly digitally connected even in the poorest countries of the world. However, we are in the early stages of understanding how to optimally design such studies.

## Abbreviations

BWW: Big White Wall
CBT: cognitive behavioral therapy
CLAHRC: Collaboration for Leadership and Applied Health Research and Care
CLAHRC-EM: Collaboration for Leadership and Applied Health Research and Care-East Midlands
CSRI: Client Service Receipt Inventory
GAD-7: 7-item Generalized Anxiety Disorder Scale
GP: general practitioner
IP: Internet protocol
MCID: minimally important clinical difference
MOS-SS: 8-item Medical Outcomes Study Social Support Survey
NHS: National Health Service
NICE: National Institute for Health and Care Excellence
NIHR: National Institute for Health Research
PHQ-9: 9-item Personal Health Questionnaire
QOL: quality of life
RCT: randomized controlled trial
RE-AIM: Reach, Effectiveness, Adoption, Implementation, and Maintenance model
SAPAS: Standardized Assessment of Personality-Abbreviated Scale
SD: standard deviation
SF-12v2: Medical Outcomes Study Short Form health survey version 2.0
WEMWBS: Warwick-Edinburgh Mental Well-Being Scale
WSAS: Work and Social Adjustment Scale
